# Narrow Complex Tachycardia Is Not Always a Supraventricular Tachycardia: A Case of Ventricular Tachycardia Presenting as a Narrow Complex Tachycardia

**DOI:** 10.7759/cureus.95789

**Published:** 2025-10-31

**Authors:** Shamsa Alkaabi, Ismail Al Abri, Hilal Al Riyami

**Affiliations:** 1 Pediatric Cardiology, Oman Medical Specialty Board, Muscat, OMN; 2 Pediatric Electrophysiology, Royal Hospital/Ministry of Health, Muscat, OMN; 3 Pediatric Cardiology, Sultan Qaboos University Hospital, Muscat, OMN

**Keywords:** ablation, children, narrow complex, tachycardia, wide-complex tachycardia

## Abstract

Ventricular tachycardia (VT) is a life-threatening arrhythmia in pediatric age. It is distinguished by a wide complex tachycardia on the electrocardiogram (ECG). Herein, we report the case of an 18-month-old toddler who presented with sudden-onset, narrow complex tachycardia at a heart rate of 240 bpm following an upper respiratory tract infection. Initial management, including multiple doses of adenosine and amiodarone, was unsuccessful. Synchronized cardioversion briefly restored sinus rhythm but was not sustained. Given the refractory nature of the tachycardia, an emergency electrophysiological (EP) study was performed, which revealed atrioventricular (AV) dissociation, confirming the diagnosis of VT despite the narrow QRS morphology. This case highlights the importance of considering VT in cases of refractory tachycardia, even when ECG findings suggest supraventricular tachycardia.

## Introduction

Ventricular tachycardia (VT) is a rare but serious life-threatening arrhythmia when it happens. Its incidence in children has been reported at around 1.1 per 100,000 [1]. It is defined as a tachycardia that originates from the ventricular myocardium. Morphologically, it can be monomorphic (identical RQS complexes with a regular rhythm) or polymorphic (several different QRS complex shapes, and the rhythm is irregular). It can present in structurally normal hearts and may lead to life-threatening events [2]. Furthermore, it can be sustained (lasting more than 30 seconds) or non-sustained (lasting less than 30 seconds). Electrocardiogram (ECG) findings suggestive of VT include wide complex tachycardia with AV dissociation, the presence of fusion or capture beats, concordance in the chest leads, and a northwest QRS axis. VT typically presents as a wide complex tachycardia because of its origin within the ventricular myocardium, so the electrical impulse bypasses the His-Purkinje conduction system and spreads through slower, myocyte-to-myocyte conduction pathways [2,3]. Subsequently, this leads to a delay in depolarization, resulting in a widened QRS complex (>120 ms).

In contrast, supraventricular tachycardia (SVT), which originates above the ventricles, typically within the atria or AV node, uses the intact His-Purkinje system for ventricular activation, creating fast yet organized and narrow QRS complexes (<120 ms) [2-4]. Though certain SVTs with aberrant conduction (e.g., bundle branch block or accessory pathways, such as in Wolff-Parkinson-White syndrome) may also display wide QRS complexes [2-4].

Early recognition and correct interpretation of ECG features are essential for effective management and favorable outcomes [5-7].

Here, we report a case of narrow complex tachycardia (NCT) that was initially diagnosed and treated as SVT but later proved to be VT. Narrow complex VT has never been reported in pediatrics. There are only a few reported cases in adults [8-10].

## Case presentation

**Figure 1:**
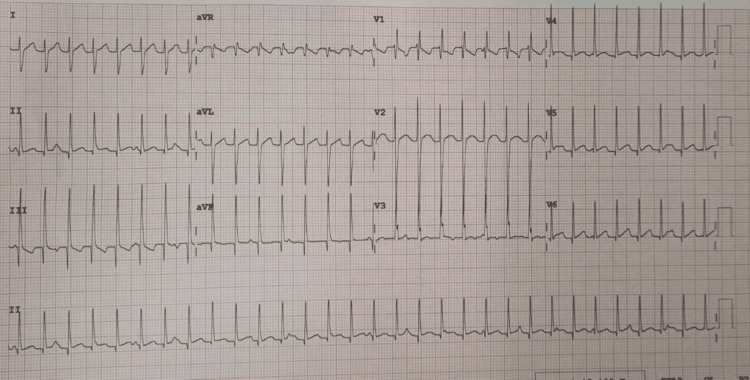
Initial ECG upon arrival at the hospital

An 18-month-old female toddler (weight 10 kg), who was previously well and healthy, developed upper respiratory tract symptoms with fever and a runny nose for two days in March 2024. On the second day of her illness, while sitting next to her mother, she suddenly began crying, became pale, and then fell down without losing consciousness. Parents took her immediately to the nearest health center, where her heart rate was documented at 240 bpm with NCT on ECG (Figure 1). Her ECG showed right-axis deviation, a normal QRS duration (<120 ms), and possible dissociation.

The patient initially received two doses of adenosine (0.05 mg/kg followed by 0.1 mg/kg) without any effect on the tachycardia. On admission to the intensive care unit, she remained in a NCT and was sedated with ketamine. Amiodarone infusion was initiated. Synchronized cardioversion was attempted with an initial dose of 0.5 J/kg, followed by 1 J/kg, which transiently restored sinus rhythm for a few minutes before recurrence of the tachycardia.

She was subsequently transferred to the cardiac unit for further evaluation and management. On arrival, she was hemodynamically stable, with an ECG showing persistent NCT similar to that shown in Figure 1. Transthoracic echocardiography revealed borderline left ventricular systolic function with an ejection fraction of 53%.

**Figure 2:**
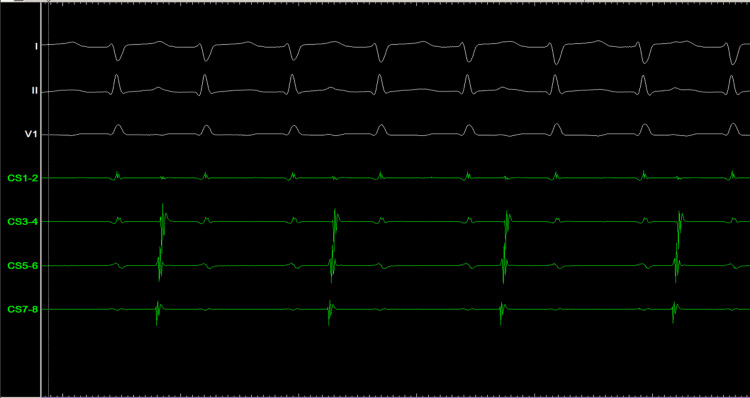
Atrioventricular (AV) dissociation in the electrophysiology (EP) laboratory

Given the incessant nature of the tachycardia and the uncertain diagnosis, an urgent electrophysiological (EP) study was undertaken. Intracardiac electrograms demonstrated clear atrioventricular (AV) dissociation (Figure 2), confirming the diagnosis of VT despite the narrow QRS morphology.

**Figure 3:**
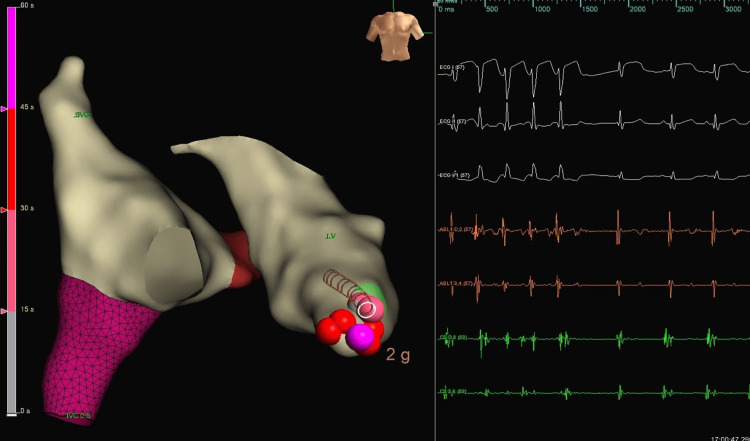
Ablation that terminated the tachycardia

The morphology was suggestive of a left-sided focus, as V1 was positive. Mapping with TactiCath and a steerable sheath showed that the earliest activation occurred in the anterior-mid septal area (Figure 3).

There was a recurrence of premature ventricular complexes (PVCs) with the same morphology, so consolidation with a maximum of 35-watt lesions was done. There was no recurrence in 45 minutes, even with an isoprenaline infusion. The amiodarone infusion was kept for 24 hours, then shifted to a maintenance dose. She was kept under observation with no recurrence of her VT. Echocardiography performed before discharge showed normal LV function with an EF of 67%, and the patient was discharged on amiodarone. During follow-up, the child remained free of arrhythmia. Amiodarone was gradually tapered and discontinued over the course of several months. After one year of follow-up, the patient was discharged in good condition. Both the ECG and 24-hour Holter monitoring showed normal findings.

## Discussion

VT is an uncommon arrhythmia in the pediatric population, particularly among children without structural heart disease or known myocardial pathology [1]. The limited availability of large-scale data on VT in otherwise healthy children contributes to the diagnostic challenge in such cases [1,2]. Atypical presentations, such as narrow complex VT or hemodynamically stable rhythms, can further obscure recognition and delay appropriate management [3].

Tachycardia in pediatrics is categorized as wide or narrow based on QRS duration [3]. If the QRS duration is less than 120 ms, it is considered narrow; if it is greater, it is considered wide complex tachycardia [4].

This child has presented with NCT, which is more common in the pediatric age group and is generally due to atrioventricular re-entry tachycardia (AVRT), atrioventricular nodal re-entry tachycardia (AVNRT), atrial tachycardia, or atrial flutter [4]. However, there was AV dissociation, and the most common diagnosis in pediatric NCT and AV dissociation is junctional ectopic tachycardia (JET) [2]. A crucial clinical feature for differentiating VT from SVT includes a lack of response to adenosine or vagal maneuvers, the presence of AV dissociation on ECG, and the occurrence of capture or fusion beats [2-4].

Nevertheless, the EP study proved that this is VT with NCT, which is very rare. In a structurally normal heart, the most common types of monomorphic VT are idiopathic infantile VT, idiopathic right ventricle outflow tract VT, and idiopathic left ventricle posterior fascicular VT [7,8]. In our case, mapping revealed a left-sided focus with the earliest activation at the anterior-mid septal area, where ablation terminated the tachycardia. Interestingly, since this child presented with NCT, he was initially misdiagnosed and managed like SVT. Sadagopan et al. reported five patients with NCT due to VT in adults, but all of them were mismanaged as SVT due to the rarity of having VT with narrow complex [9].

Only a few cases of NCT with AV dissociation have been reported in young patients without ischemic heart disease [10,11]. However, it is more commonly seen in adults post-cardiac infarct [9]. Bostan et al. demonstrated that Purkinje fibers could be responsible for NCT arising from the ventricle, particularly after an ischemic insult [11].

In the pediatric age group, we should always consider myocarditis, as ischemia and fibrosis can trigger such arrhythmias [12]. Interestingly, this child has no features suggestive of myocarditis.

## Conclusions

This case highlights that an NCT unresponsive to conventional SVT therapies may, in fact, represent an atypical presentation of VT. Such cases warrant a high index of suspicion and thorough evaluation, including advanced diagnostics such as EP studies, to ensure accurate diagnosis and guide appropriate management.
